# Induction of Osseointegration by Nacre in Pigs

**DOI:** 10.3390/molecules27092653

**Published:** 2022-04-20

**Authors:** Leena Leelatian, Panjit Chunhabundit, Phingphol Charoonrut, Pattapon Asvanund

**Affiliations:** 1 Ph.D. Student, Molecular Medicine Program, Faculty of Science, Mahidol University, Bangkok 10400, Thailand; l.leelatian@gmail.com; 2 Department of Anatomy, Faculty of Dentistry, Mahidol University, Bangkok 10400, Thailand; 3 Department of Clinical Science and Public Health, Faculty of Veterinary Science, Mahidol University, Nakhon Pathom 73170, Thailand; phingphol.cha@mahidol.edu; 4 Independent Researcher, Bangkok 10400, Thailand; pat.dds@hotmail.com

**Keywords:** bone regeneration, dental implant, nacre, osseointegration

## Abstract

Nacre is a biomaterial that has shown osteoinductive and osteoconductive properties in vitro and in vivo. These properties make nacre a material of interest for inducing bone regeneration. However, information is very limited regarding the introduction of nacre to dental implant surgery for promoting osteogenesis. This study investigated the potential of nacre powder for peri-implant bone regeneration in a porcine model. Ninety-six dental implants were placed into the tibia of twelve male domestic pigs. The dental implants were coated with nacre powder from the giant oyster before implantation. Implantations without nacre powder were used as control groups. Euthanization took place at 2, 4 and 6 weeks after implantation, after which we measured bone-to-implant contact (BIC) and bone volume density (BVD) of the implanted bone samples using micro-computed tomography (micro-CT), and examined the histology of the surrounding bone using histological sections stained with Stevenel’s blue and Alizarin red S. The micro-CT analyses showed that the BIC of dental implantations with nacre powder were significantly higher than those without nacre powder, by 7.60%. BVD of implantations with nacre powder were significantly higher than those without nacre powder, by 12.48% to 13.66% in cortical bone, and by 3.37% to 6.11% in spongy bone. Histological study revealed more peri-implant bone regeneration toward the surface of the dental implants after implantation with nacre powder. This was consistent with the micro-CT results. This study demonstrates the feasibility of using nacre to promote peri-implant bone regeneration in dental implantation.

## 1. Introduction

Tooth loss is a health problem that affects millions of people worldwide. This issue can be an aesthetic concern associated with psychological well-being and quality of life. The placement of osseointegrated implants has become an efficient and well-accepted method of replacing lost teeth. For successful implant treatment, one of the most important determinants is the bone-implant interface. When the surface of an implant is directly connected to living bone tissue and remains stable during functional loading, this phenomenon involves wound healing of surrounding bone tissue and subsequent remodeling as well as bone formation along the implant surface [1,2]. With the goal of providing better healing around dental implants, various implant systems and modifications have been developed to aid the mechanism of osseointegration in speeding up and strengthening new bone formation. Macrotopography of dental implants plays a major role in the primary stability and the mechanical engagement of an implant with the surrounding bone after implant integration. This initial stability is critical for secondary implant stability, which is a biological phenomenon. The microscopic features of dental implants are situated on surfaces treated with techniques such as acid etching, sand blasting, grit blasting, hydroxyapatite coating, and anodization, with a scale range of microroughness from 1 to 100 microns. Modifications create roughness on the surface, which increases the surface area to facilitate bone growth, turnover and remodeling [3,4,5,6]. Studies have shown better outcomes and more bone-to-implant contact on rough surfaces than on smooth surfaces [7,8]. The degree of surface roughness is positively correlated with bone-to-implant contact. Ivanoff et al. [9] demonstrated that the bone-to-implant contact of TiO_2_ blasted microimplants was significantly higher than that of machined microimplants. In addition, more bone was observed in the threaded area in blasted microimplants than in the smoother surface of machined microimplants. Recently, nanotechnology has been introduced to modify the surfaces of dental implants. It has been proposed that roughness at a nanoscale range (1–100 nanometers) positively affects osteoblast-specific gene expression, and the proliferation and differentiation of osteoblastic cells [10,11]. Moreover, implants coated with growth factors, bone-related proteins, and bioactive agents have been found to be effective in bone formation [12]. Bone morphogenic proteins (BMPs) loaded on the surface of implants have also been found to induce bone formation [13].

Nacre is the innermost layer of mollusk shells. This composite natural material consists of up to 95 wt% calcium carbonate in aragonite crystal form, and 1 to 5 wt% organic matrix that forms the rest of nacre, mostly made of proteins, glycoproteins, and polysaccharides. [14]. The osteogenic properties of nacre have previously been reviewed [15,16]. To date, many of its proteins have been successfully purified and identified [15]. It has been proposed that nacre contains signaling molecules involved in the process of bone formation. Previous studies have demonstrated that nacre has osteoinductive properties for mineralization in vitro [17,18,19]. Nacre powder as a bone graft material has been shown to induce new bone formation in vivo. Pulverized nacre mixed into a slurry with blood was implanted into human jaw bone defects and was capable of inducing bone regeneration. New bone was formed throughout the implanted material [20]. Other studies have also shown that nacre is a biocompatible, biodegradable and osteoinductive material, and can initiate bone formation in vivo [21,22,23,24,25,26,27]. Liao et al. [22] implanted nacre granules (<2 mm diameter) into rat femurs. New healthy bone was formed directly on the nacre surface, with a phosphorous-rich zone in the interface between bone and the implants. Biocompatibility, biodegradability and osteoinductive properties make nacre a promising candidate for facilitating the mechanism of osseointegration. However, in vivo information on osseointegration in dental implants induced by nacre is very limited. This study aimed to demonstrate osseointegration in domestic pigs after dental implant installation in conjunction with nacre.

## 2. Results

In this study, specimens from 12 experimental animals were analyzed. Evaluation was performed of bone tissue response to dental implants with and without nacre powder, through micro-CT analysis. Reconstructed 3D images of peri-implanted bone were created. Histological sections of these specimens were subsequently studied to evaluate bone formation in the peri-implant area.

### 2.1. Micro-CT Imaging

**Figure 1:**
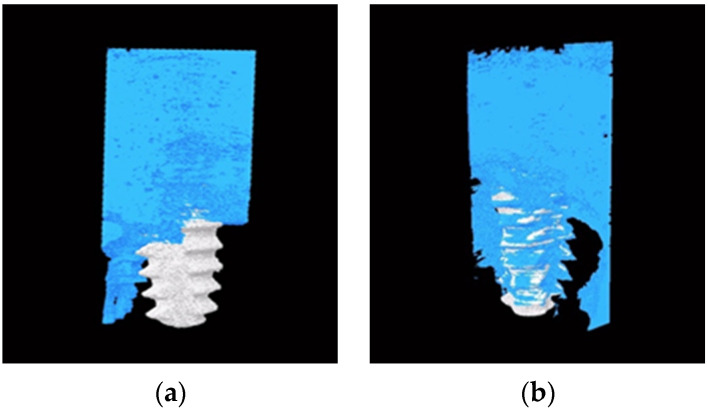
The reconstructed 3D images showing dental implants (white) and peri-implant bone tissue (blue) after 4 weeks of implantation. (**a**) Implant placement without nacre powder. (**b**) Implant placement with nacre powder.

Reconstructed 3D images of bone architecture surrounding the implant without and with nacre powder, 4 weeks after implantation, are revealed in Figure 1a,b, respectively. The dense cortical bone covers most of the upper half of the implants in both groups. Spongy bone, characterized by mesh-like networks that intersperse in the bone marrow, is more frequently found along the surface of implant with nacre powder (Figure 1b).

### 2.2. Micro-CT Analysis

#### 2.2.1. Bone-to-Implant Contact (BIC)

**Figure 2:**
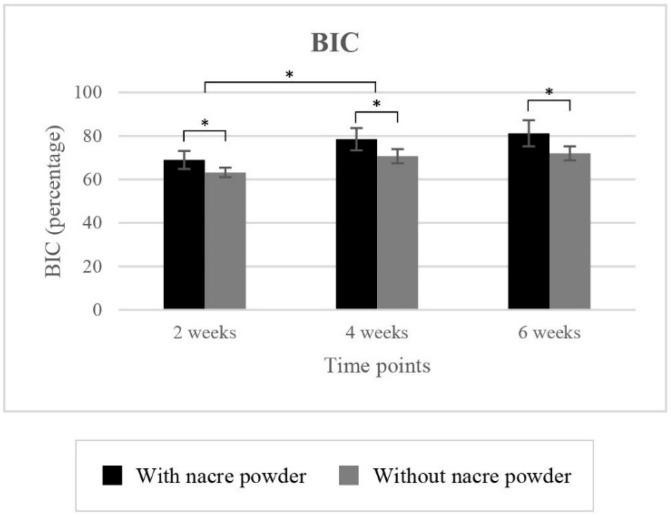
The clustered columns showing mean BIC in percentage of implantations with or without nacre powder using XiVE^®^ dental implants. * indicates difference at significance level 0.05.

Mean BIC values for implantation with and without nacre powder are shown in Table 1 and Figure 2. Statistically, implantation with nacre powder had a significantly higher BIC (*p* < 0.001), a 7.60% increase compared to implantation without nacre powder. Significant differences in BIC were found between weeks 2 and 4 (*p* < 0.001), but not between 4 and 6 weeks (*p* = 0.150). 

#### 2.2.2. Bone Volume Density (BVD)

##### BVD of Cortical Bone

**Figure 3:**
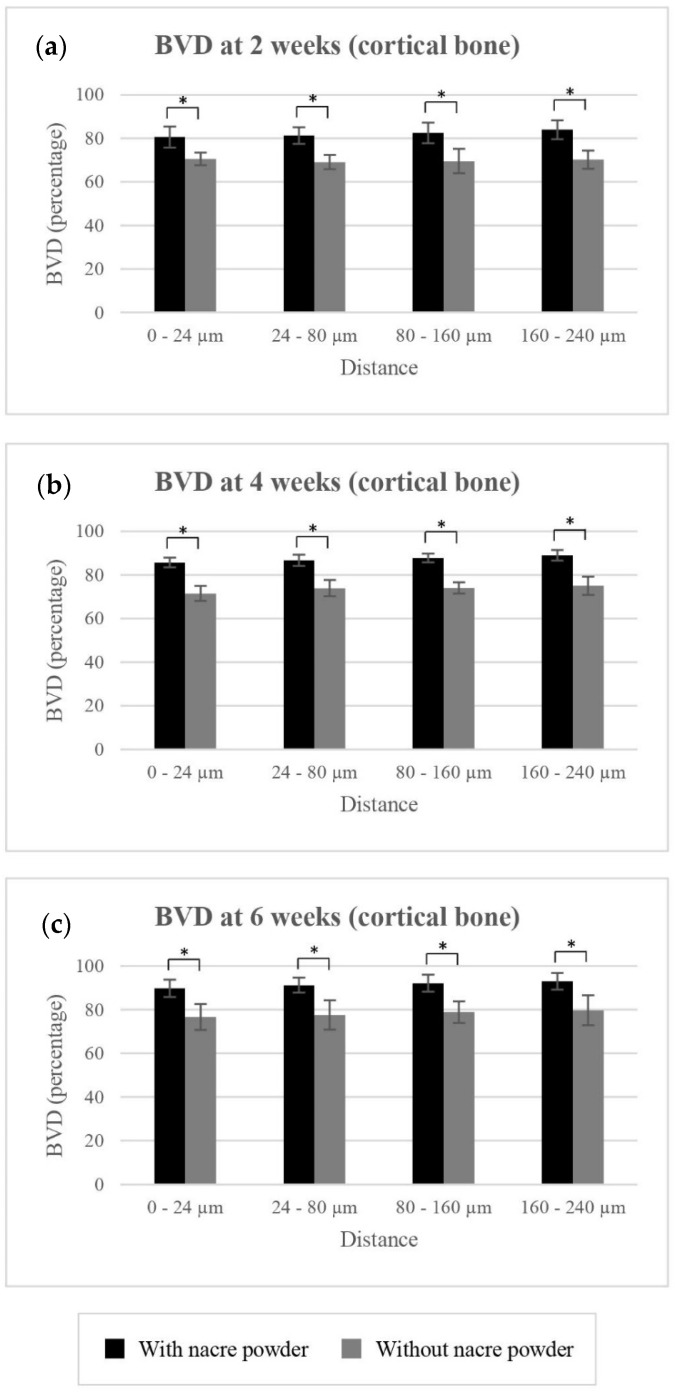
The clustered columns showing mean BVD in percentage of implantations with or without nacre powder, using XiVE^®^ dental implants in cortical bone at distances of 0–24 µm, 24–80 µm, 80–160 µm, and 160–240 µm from the implant surface. (**a**) At week 2. (**b**) At week 4. (**c**) At week 6. * indicates difference at significance level 0.05.

Mean BVD values for cortical bone surrounding implantation with and without nacre powder are shown in Table 1 and Figure 3. Implantation with nacre powder resulted in significantly higher BVD of cortical bone at distances of 0–24 µm (*p* < 0.001), 24–80 µm (*p* < 0.001), 80–160 µm (*p* < 0.001), and 160–240 µm (*p* < 0.001) from the implant surface, an increase compared to controls by 12.48%, 12.86%, 13.31%, and 13.66%, respectively. Significant differences of BVD were found between weeks 2 and 4 (*p* < 0.001) and between 4 and 6 weeks (*p* = 0.00) at every distance measured. These data suggest the use of nacre to promote cortical bone regeneration in the peri-implant area.

##### BVD of Spongy Bone

**Figure 4:**
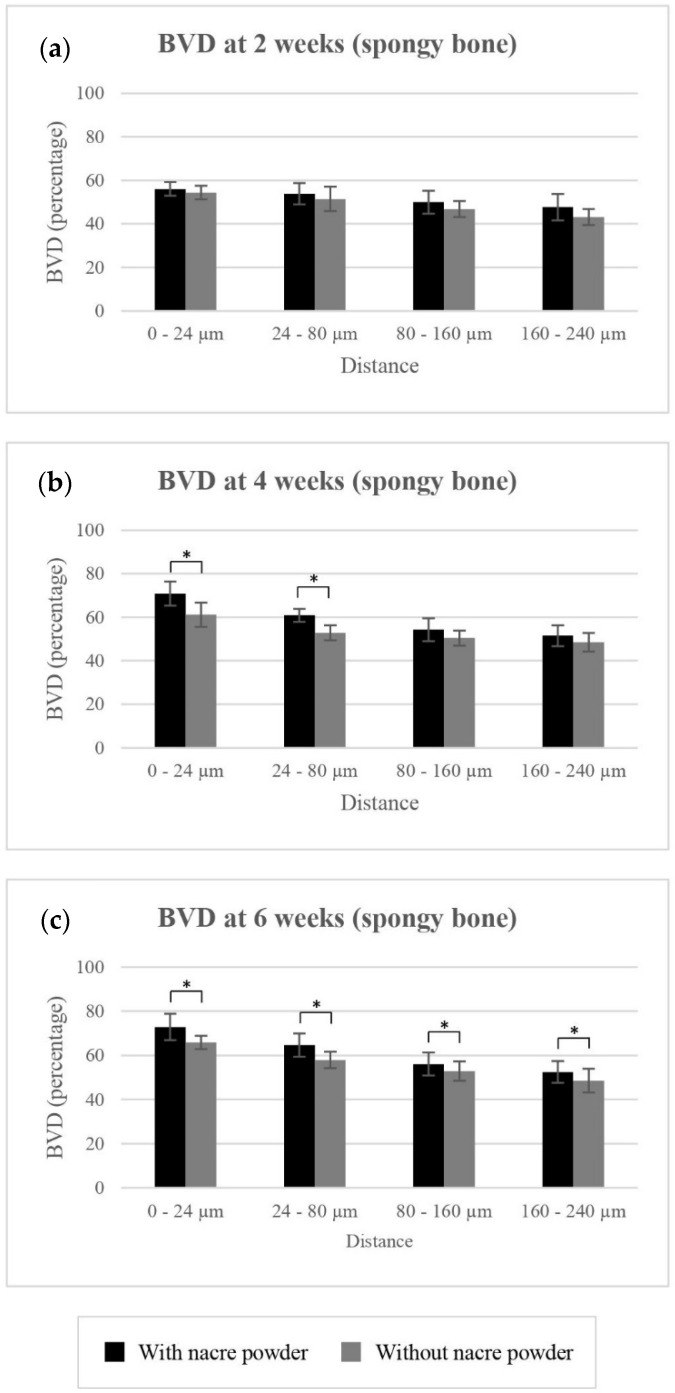
The clustered columns showing mean BVD in percentage of implantations with or without nacre powder, using XiVE^®^ dental implants in spongy bone at distances of 0–24 µm, 24–80 µm, 80–160 µm, and 160–240 µm from the implant surface. (**a**) At week 2. (**b**) At week 4. (**c**) At week 6. * indicates difference at significance level 0.05.

Mean BVD values of spongy bone surrounding implantation with and without nacre powder are shown in Table 1 and Figure 4. Implantation with nacre powder resulted in significantly higher BVD percentages of spongy bone, compared to implantation without nacre powder, at distances of 0–24 µm (*p* < 0.001), 24–80 µm (*p* < 0.001), 80–160 µm (*p* = 0.018), and 160–240 µm (*p* = 0.018) from the implant surface, an increase of 6.11%, 5.74%, 3.37%, and 3.78%, respectively.

At distances of 0–24 µm, 80–160 µm and 160–240 µm, significant differences were found between weeks 2 and 4 (*p* ≤ 0.001), but not between 4 and 6 weeks (*p* ≥ 0.05). However, at distances of 24–80 µm, significant difference in BVD was not observed between weeks 2 and 4 (*p* = 0.051) but was found between 4 and 6 weeks (*p* = 0.001). These results indicate the positive effect of nacre on spongy bone regeneration. 

### 2.3. Histological Study

**Figure 5:**
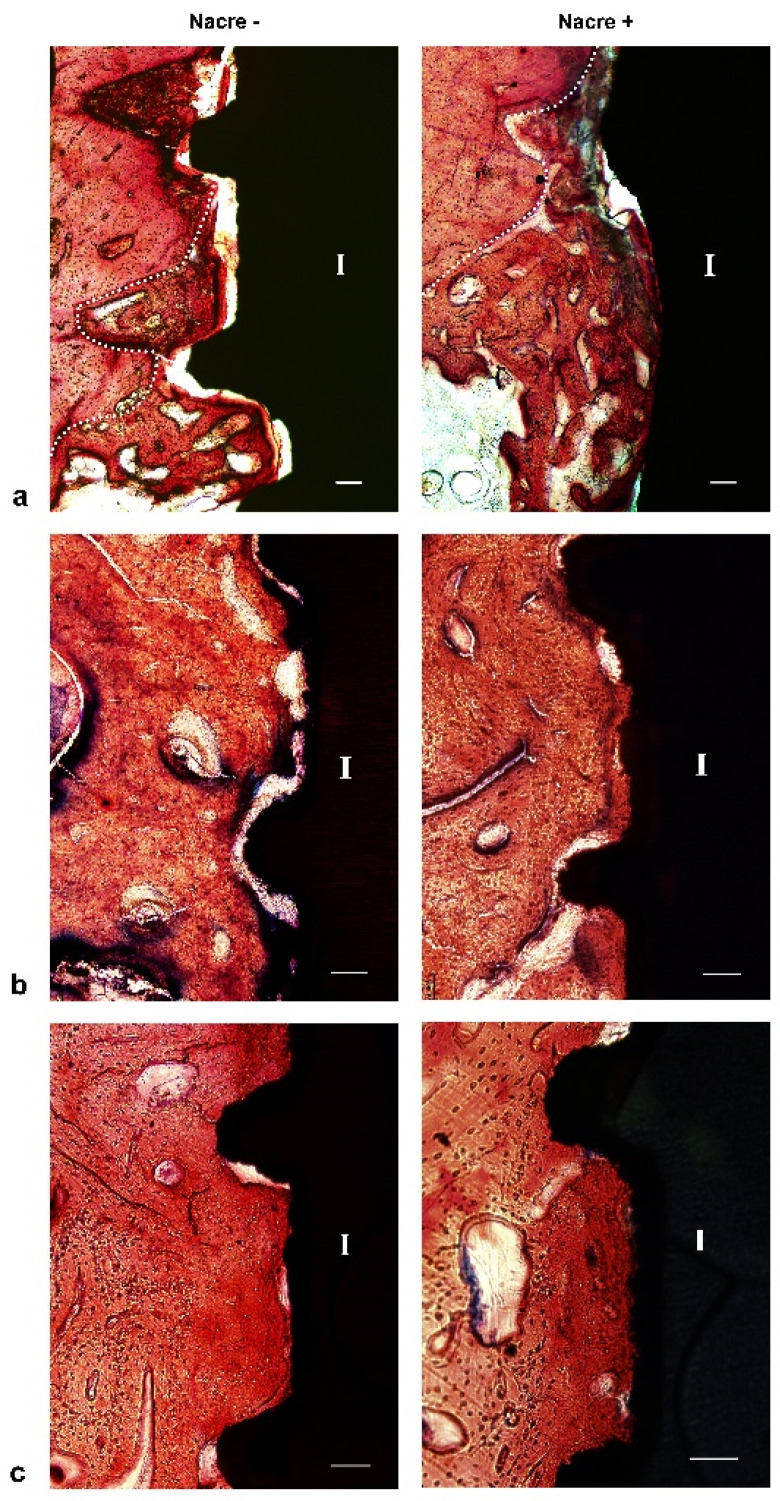
Light micrographs of XiVE^®^ implant longitudinal sections treated without (left panels) or with (right panels) nacre powder. (**a**) At week 2. (**b**) At week 4. (**c**) At week 6. The dotted line indicates the interface between the old and new bone; I, implant. Bar = 100 µm. Stevenel’s blue and Alizarin red S stain.

**Figure 6:**
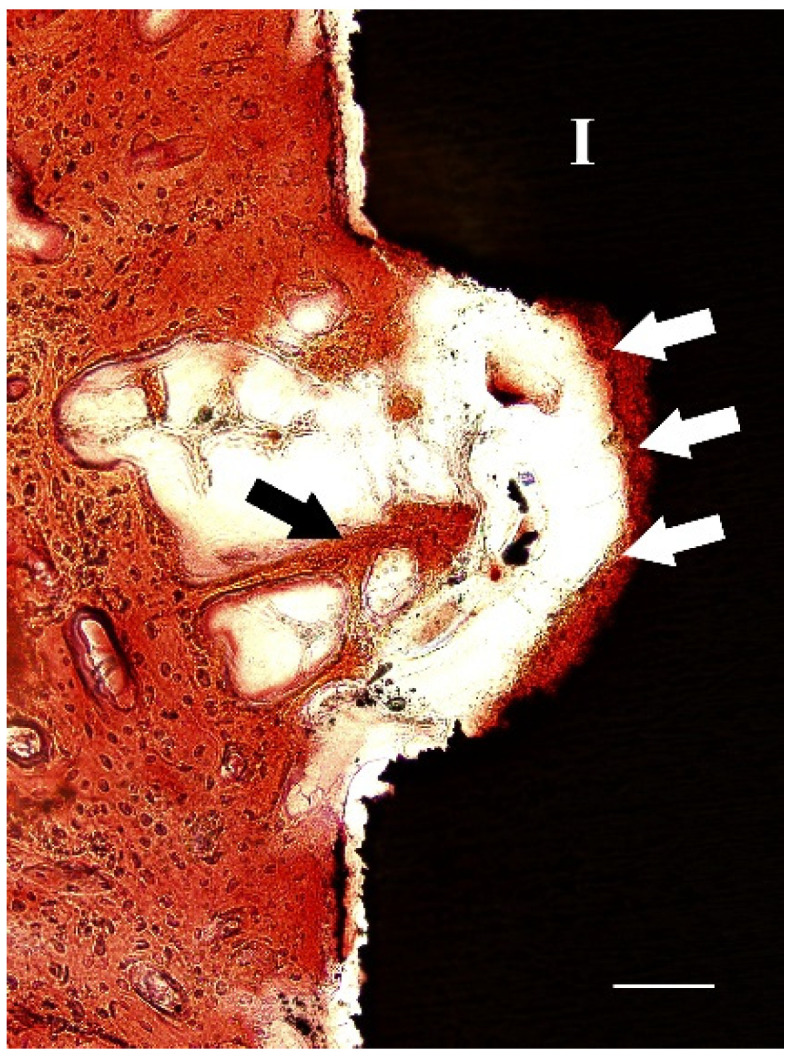
A light micrograph of a section of XiVE^®^ implant after 4 weeks of implantation with nacre powder, showing an area of mineralized tissue with bone cells (white arrows) attached directly to the implant surface. Trabeculae (black arrow) were found approaching the implant surface; I, implant. Bar = 100 µm. Stevenel’s blue and Alizarin red S stain.

After two weeks of implantation, histological sections revealed that irregular and disorganized orientations of trabeculae resembling woven bone were formed close to the marrow space and towards the dental implants (Figure 5a). These newly formed trabeculae were more frequently observed after implantation with nacre powder than after implantation without nacre powder. At four weeks after implantation (Figure 5b), woven bone in contact with the implant surface was frequently found in histological sections of dental implants with nacre powder, rather than those without nacre powder. Non-mineralized matrices stained light green-blue were seen in sections of the implantation without nacre powder more than in implantation with nacre powder. It is worth noting that a thin layer of calcified tissue stained with Alizarin red S was found deposited directly on the surface of dental implants with nacre powder. Some bone cells were seen within this tissue (Figure 6). At six weeks after implantation (Figure 5c), it was shown that bone tissue at the site of implantation with nacre powder was in more continuous contact with the dental implant surface than at the site of implantation without nacre powder. Moreover, the circular arrangement of bone cells was more regular between weeks 2 and 4. The results of histochemical staining were consistent with those of micro-CT analyses.

## 3. Discussion

In this study, the effects of nacre powder on osseointegration in vivo were investigated. Domestic pigs were used because their rates of bone formation (1.2–1.5 µm/day) are most similar to those of humans (1.0–1.5 µm/day) [28,29]. Quantitative evaluation of bone tissue response through micro-CT imaging was conducted to study the osseointegration of dental implants, as its analysis is fast, non-destructive, and allows 3-dimensional evaluation. Butz et al. [30] compared the correlation between micro-CT and histological imaging for cortical and cancellous bones at distances of 0–24 µm, 24–80 µm, 80–160 µm and 160–240 µm from the implant surface. The study found that the bone configuration in micro-CT images corresponded to that observed in histologic sections. Nevertheless, the correlation between micro-CT and histology was significant for cortical and cancellous bone at 24–240 µm from the implant surface, but was not significant at a distance of 0–24 µm from the surface. In the present study, micro-CT analysis showed that nacre powder increased the mean BIC percentages at every time point. When measuring BVD in cortical bone, our findings suggested that implantation with nacre powder resulted in significantly higher mean BVD at every time point and in every measured range of distances from the implant surface (0–24 µm, 24–80 µm, 80–160 µm and 160–240 µm). At distances further away from the implant surface, mean BVDs of implantations with and without nacre powder were both increased. It is noteworthy that the differences in mean BVD between implantation with and without nacre powder at every measured distance from the implant surface in cortical bone were much greater than those in spongy bone. Moreover, the mean BVDs of implantations with and without nacre powder were both decreased at distances further away from the implant surface. This may be because spongy bone is made up of trabecular tissue mesh interspersed between bone marrow. At distances further away from the implant surface, nacre powder coated on the surface of dental implants prior to installation may have gradually dissolved and diluted into the surrounding tissue.

In this present study, histologic ground sections of the implant and surrounding bone tissue were examined using Alizarin red S and Stevenel’s blue staining after two, four and six weeks of implantation. The positive staining of Alizarin red S and Stevenel’s blue were clearly detected as orange-red and green-blue areas, respectively. This result is supported by previous published studies. A thin layer of calcified tissue stained with Alizarin red S found in this study deposited directly on the implant surface of the nacre powder-presenting group but not the control group is similar to that reported by Marco et al. [31]. The authors demonstrated that the sand-blasted surface of titanium dental implants inserted in sheep femur was widely covered by a thin layer of calcified tissue directly on the implant surface, suggesting contact osteogenesis. Interestingly, Puleo and Nanci [32] reported that contact osteogenesis is 30% faster than distance osteogenesis. Contact osteogenesis may better contribute to the development of biological implant fixation as new bone directly forms on the implant surface. In addition, after four weeks of implantation with nacre powder, trabecular bone was found close to the implant surface, suggesting distance osteogenesis. This trabecular bone is particularly suitable for the implant healing process, as it shows a very active wide surface area contiguous with marrow spaces, including many vessels and mesenchymal cells [33].

Lamghari et al. [23] used nacre powder, sized 50–150 µm, to fill cavities in the upper lumbar region of sheep. The results showed that the newly formed bone was in close contact with the nacre implant. The degree of mineralization of the bone adjacent to cavities filled with nacre increased when compared to empty cavities. Nacre particles that were in contact with the trabeculae were gradually dissolved between weeks 1 and 8. The results of the present study agree with those of Lamghari et al. [23]. Although nacre powder is resorbed gradually, it has shown the capability of promoting new bone formation. The nacre powder was fused into the existing bone and could not be clearly identified. Thus, it is possible that the nacre powder induced bone formation and might be replaced by the newly formed bone, which becomes welded together with the pre-existing bone. This result was in agreement with the findings of our previous study, that nacre particles were resorbed and replaced by bone after 30 days of nacre rod implantation in the mandibles of guinea pigs [27]. 

In recent decades, the biological properties of nacre have been studied. Water soluble matrix (WSM) extracted from nacre has been demonstrated to enhance bone-related gene expression, osteoblastic activity, and bone mineralization [34]. In an in vitro study carried out by Asvanund et al. [18], nacre chips induced the expression of alkaline phosphatase at weeks 2 and 4 and the expression of bone sialoprotein at weeks 3 and 4 in cultured human bone cells. These results suggest that nacre has osteogenic effects mostly at the phase of extracellular matrix maturation. However, osteocalcin gene expression was not elevated by nacre at week 2, and was not different from that of beta-tricalcium phosphate (β-TCP) at weeks 3 and 4. This finding suggests that nacre has an osteogenic effect at the phase of extracellular matrix mineralization comparable to that of β-TCP.

Limited studies are currently available regarding the mechanism of bone regeneration which nacre influences. Recent evidence of such mechanisms was reported in a study by Cheng Y et al. [35]. The authors investigated the role of water-soluble nano-pearl powder (WSNNP) on osteoblast differentiation and its underlying mechanisms. The study found that WSNNP may contribute to osteoblast differentiation by enhancing autophagy via the MEK/ERK signaling pathway. Furthermore, many bioactive molecules present in nacre have been characterized [12], including Lustrin A, nacrein, Perlucin, Perlustrin, MSI31, M SI60 and N16. These proteins play important roles in the formation of aragonite crystal for shell formation. Such proteins may be involved in the regulatory mechanisms of osteogenesis. These results may suggest the additional potential of nacre as a biological material for use in bone implants.

## 4. Materials and Methods

### 4.1. Implanted Materials

#### 4.1.1. Nacre Powder

The nacreous layer (nacre) of the giant oyster (*Pinctada maxima*) shell was pulverized using a centrifuge crusher (Retsch S1000F; Kurt Retach GmbH & Co. KG, Hasloch, Germany) and sieved to obtain particle sizes < 100 µm using a vibrator sieve shaker (Pro Analysette 3; Fritsch, Ider-Oberstein, Germany). The nacre powder was sterilized by autoclave (HA-300T; Hirayama, Saitama, Japan).

#### 4.1.2. Dental Implants

Total of 96 threaded titanium implants (XiVE^®^; Dentsply Sirona, Charlotte, NC, USA), 4.5 mm in diameter and 8 mm in length, were used.

### 4.2. Animal Implantation

Twelve male domestic pigs (*Sus domesticus*) of four months of age, weighing approximately 40 kg, supplied by Somboon Farm, Thailand, were used. The experimental design was approved by the Animal Care and Use Committee, Faculty of Veterinary Science (approval number MUVS-2-12-52). The experiment was conducted at the Animal Operation Room, Faculty of Veterinary Science. The animals were randomly assigned to three groups; thus, four animals were studied in each group.

**Figure 7:**
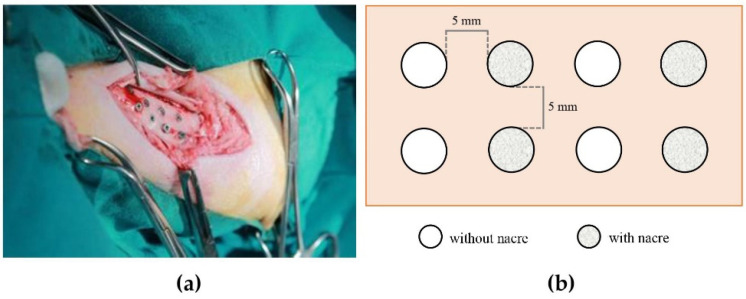
(**a**) A photograph showing dental implants surgically placed in a tibia. (**b**) Schematic drawing of dental implant placement design in the tibia of each animal.

All animals were nil per os for 12 hours before surgery and sedated with an intramuscular injection of 5 mg/kg tiletamine/zolazepam (Zoletil^®^) compound. General anesthesia was maintained with an inhalant agent (isoflurane). The level of anesthesia was checked before the surgical procedure. The skin on the left tibia of the hind leg was shaved and cleaned with antiseptics, then the proximal tibial epiphysis was located by palpation. The incision was made along the tibial diaphysis, and the flaps were raised. A round-shaped carbide bur was used as an initial drill. Then, a 2.0 mm twist drill was drilled further to an 8 mm depth, followed by a 3.0 mm twist drill, and a pre-tap drill to an 8.0 mm depth. Drilling was performed under normal saline irrigation. Eight implants were placed in one tibia of each animal. With the use of a custom-made template, each implant was positioned approximately five millimeters apart. Four implants were assigned for implant placement with nacre powder as experimental samples whereas the other four implants without nacre powder were control samples (Figure 7). For implantation with nacre, each dental implant was submerged in a mixture of 0.1 g of sterile nacre powder and the animal’s own blood prior to installation into the prepared socket. All implants were surgically placed according to manufacturer protocol. All implants were randomized to the treatment groups and implantation sites on the tibia. Flaps were tension released and stitched with Vicryl^®^ 3-0 to obtain primary closure. All animals were administered antibiotic and analgesic drugs for seven days after the surgery, and were housed at the Faculty of Veterinary Science laboratory animal room.

The animals were euthanized at two, four and six weeks after surgery, by intravenous overdosing with 20 mg/kg pentobarbital sodium and ten milliliters of potassium chloride (20 mEq./10 mL). Four animals were randomly assigned to each euthanizing time point. The specimens were excluded if the animal died prematurely or if the implant was displaced from the surgical site. All specimens from each animal, including the bone, implants, and surrounding tissues, were collected *en bloc* and preserved in 10% neutral buffered formalin solution at 4 °C for 48 h. The bones were cut with a low-speed diamond saw (IsoMet; Buehler, Lake Bluff, IL, USA), leaving at least five millimeters of bone surrounding the implants. The size of the resulting bone specimens was approximately W 15 mm × L 30 mm × H 20 mm.

### 4.3. Micro-CT Study

The specimens were washed thoroughly with 0.1 M phosphate-buffered saline solution, pH 7.4, before being analyzed using SkyScan 1173 (Bruker, Kartuizersweg 3B, Kontich, Belgium). Scans were performed at an X-ray level of 130 kV, 61 µA and 0.25 mm Brass filter. The image pixel size was 8.10 µm, 0.2 degree rotation step, 360 degrees scan rotation, and 1300-ms exposure time. All data were imported into NRecon software (v. 1.6.8.0, Bruker micro-CT) for reconstruction. The volume-of-interest (VOI) data were analyzed with CT-Analyzer software (CTAn, v. 1.13.5.1, Bruker micro-CT). The four voxels closest to the screw’s surface were excluded to eliminate a possible artefact zone. BIC, defined as the percentage area of the total implant surface covered by bone, was calculated by 2D analysis. The percentages of peri-implant bone volume density (BVD) were calculated separately for cortical and spongy bones. BVD was calculated at four consecutive distances measured from the implant surface: 0–24 µm, 24–80 µm, 80–160 µm and 160–240 µm. 3D images of peri-implant bone were reconstructed with CT-Voxel software (CTVol, v. 2.3.2.0, Bruker micro-CT).

### 4.4. Histological Study

The specimens were washed with 0.1 M phosphate-buffered saline solution at a pH of 7.4, dehydrated through a graded series of ethanol (70%, 80%, 95% and 100% ethanol), and embedded in a Technovit^®^ 9100 methyl methacrylate (Kulzer GmbH, 61,273 Wehrheim, Germany) block. Each specimen block was cut through a long axis of the implant by a low-speed diamond saw, resulting in two to four sections of approximately 400 µm thick. All sections were glued to plastic slides, ground with sandpaper at decreasing grits from 800 to 3200, and finally polished with an EXAKT grinding system (Exakt Company, Hamburg, Germany) to obtain 20–30 µm thick sections. Then, the sections were stained with Stevenel’s blue and Alizarin red S, which gave two separate colors for connective tissues and mineralized tissues, respectively. The sections were observed and photomicrographed under a BX53 Olympus light microscope (Olympus, Tokyo, Japan).

### 4.5. Statistical Analysis

Mean values and standard deviations were calculated for all parameters evaluated. The mean difference and standard deviation between the different groups were analyzed by comparing means. The generalized estimating equation (GEE) was applied to evaluate the data from each treatment group, different time points, and controls. For all statistical analyses, a significance level of 5% was adopted.

## 5. Conclusions

Nacre powder can promote peri-implant osteogenesis in the tibias of domestic pigs. This bioactive material stimulated more bone formation around the implant surface than was present in control groups, as observed by both micro-CT analysis and histological study. The feasibility of using nacre powder in conjunction with surgical implant placement appears to be an alternative method to promote osseointegration.

## Figures and Tables

**Table 1:** BIC and BVD percentages of implantation without and with nacre powder at different time points.

		Treatment					
	Without Nacre Powder (Controls)				With Nacre Powder		
Time Points	2 Weeks	4 Weeks	6 Weeks		2 Weeks	4 Weeks	6 Weeks
	BIC (%)						
	63.20 ± 2.20	70.67 ± 3.23	71.99 ± 3.24		68.99 ± 4.13	78.47 ± 5.15	81.19 ± 5.96
	BVD (%): cortical bone						
0 to 24 µm	70.52 ± 2.91	71.45 ± 3.42	76.62 ± 5.97		80.61 ± 4.78	85.62 ± 2.18	89.79 ± 3.94
24 to 80 µm	69.09 ± 3.24	73.88 ± 3.77	77.62 ± 6.71		81.27 ± 3.75	86.68 ± 2.63	91.21 ± 3.39
80 to 160 µm	69.56 ± 5.64	74.01 ± 2.60	78.92 ± 4.95		82.49 ± 4.75	87.78 ± 1.97	92.14 ± 3.92
160 to 240 µm	70.22 ± 4.22	75.05 ± 4.19	79.71 ± 6.85		83.96 ± 4.36	88.99 ± 2.41	93.01 ± 3.77
	BVD (%): spongy bone						
0 to 24 µm	54.36 ± 3.14	61.12 ± 5.54	65.84 ± 3.02		56.02 ± 3.10	70.81 ± 5.49	72.83 ± 6.01
24 to 80 µm	51.46 ± 5.52	52.79 ± 3.45	57.89 ± 3.72		53.85 ± 4.84	60.86 ± 2.98	64.64 ± 5.33
80 to 160 µm	46.80 ± 3.72	50.45 ± 3.43	52.87 ± 4.40		49.95 ± 5.27	54.23 ± 5.30	56.05 ± 5.18
160 to 240 µm	43.15 ± 3.71	48.50 ± 4.24	48.54 ± 5.30		47.65 ± 6.03	51.47 ± 4.86	52.42 ± 4.89

Values are presented as mean ± standard deviation. BIC: Bone-to-implant contact, BVD: Bone volume density.

## Data Availability

The data presented in this study are available on request from the corresponding author.

## Sample Availability

Samples of the compounds are available from the authors.
